# Metabolic profiles differences of overweight patients on olanzapine, clozapine and risperidone

**DOI:** 10.1192/bjo.2021.663

**Published:** 2021-06-18

**Authors:** Siew Fai Liew, Gupta Bhanu, Jimmy Lee, Kok Seng Chee, Li Qi Neo, Charmaine Tang

**Affiliations:** Institute of Mental Health, Singapore

## Abstract

**Aims:**

We set out to examine the differences in metabolic profiles of at risk (overweight) patients across commonly used atypical antipsychotics (Olanzapine, Risperidone, Clozapine). We hypothesized that Olanzapine and Clozapine group will have more metabolic abnormalities compared to Risperidone.

**Background:**

Cardiovascular diseases remain the leading cause of morbidity and mortality among people with schizophrenia. Since the introduction of atypical antipsychotics, there is cumulative evidence of their association with metabolic abnormalities. Clozapine and Olanzapine are known to constitute the highest metabolic risks amongst atypical antipsychotics.

**Method:**

This study is based on the data of 67 subjects recruited into a 12-week open-labelled trial looking at the effects of adjunctive Aripiprazole in atypical antipsychotics for weight reduction and improvement in metabolic profile. Metabolic profiles including weight, waist circumference, fasting blood glucose, HbA1c, serum total, HDL and LDL cholesterol levels and triglycerides were measured at baseline. The measurements were then compared across the different subgroups of atypical antipsychotics. The definition of metabolic syndrome proposed by the Third Report of the National Cholesterol Education Program Expert Panel (Adults Treatment Panel III) was used.

**Result:**

The atypical antipsychotics were grouped into Olanzapine (n = 27), Risperidone (n = 24) and Clozapine (n = 16). More than 50% of clozapine-treated and Olanzapine-treated overweight patients were demonstrated to have metabolic syndrome at baseline. There was a statistically significant difference in serum triglycerides (p = 0.012), LDL (p = 0.046) and HbA1c (p = 0.045) across the three groups as demonstrated by one-way ANOVA. A Tukey post hoc test showed that both the Olanzapine (p = 0.032) and Risperidone (p = 0.013) groups demonstrated statistically significant lower serum triglycerides when compared to Clozapine. Interestingly, the mean serum HbA1c was significantly lower in Clozapine when compared to Olanzapine group (p = 0.045), perhaps reflecting the closer monitoring of fasting blood sugar in clozapine patients. When controlled for age and BMI, the significant differences in serum triglycerides remain between Clozapine and Risperidone groups [but not for serum HbA1c]. There were no statistically significant differences across the groups with respect to other metabolic parameters.

**Conclusion:**

At baseline, metabolic dysregulation was demonstrated in all subgroups of overweight patients. As hypothesized, patients on Olanzapine and Clozapine groups fared worse than Risperidone. Further studies examining long term effects of atypical antipsychotics in a larger sample of patients are warranted to confirm these findings. These findings have clinical significance in terms of choosing the first antipsychotic for drug naïve patients or where there is no clinically significant difference in efficacy.

